# Achieving a time to first consultant review of under 12 hours for acutely ill medical patients

**DOI:** 10.1186/cc14589

**Published:** 2015-03-16

**Authors:** B Greatorex, EC Colley

**Affiliations:** 1 Great Western Hospital, Swindon, UK

## Introduction

In 2007, the Acute Medicine Task Force made recommendations about the operation and staffing of acute medical units (AMU). Consultant-led care was seen as critical to ensuring high standards of care for patients and maintaining efficient patient flow [1]. It also recommended that during the hours when the AMU is staffed by a consultant, all new patients should be seen within 6 to 8 hours. Patients admitted overnight should have a consultant review within 12 to 14 hours. Following the Introduction of a 4:00 pm consultant ward round of newly admitted acute medical patients to the existing 8:00 am and 2:00 pm rounds, it was our intention to establish whether our trust was meeting those recommendations.

## Methods

We conducted a prospective survey of all new acute medical admissions over a 2-week period. Data collected included date and time of admission to the hospital, location on arrival, time of first medical clerking, and time of first consultant review.

## Results

Data were collected for 420 admissions. Sixty-seven percent of patients were admitted to the hospital between 12:00 am and 12:00 pm with a peak occurring between 4:00 pm and 6:00 pm. Sixty-two percent of patients were first seen by a consultant within 12 hours of admission, with a range from 23 minutes to 26 hours. When looking at patients admitted during the weekdays, 63% of them were seen within 12 hours; for those admitted at the weekend the figure was 57%.

## Conclusion

In 2011 the Royal College of Physicians emphasized the impact that the quality of the care provided within the first 48 to 72 hours had on clinical outcomes. An evaluation of consultant input into acute admissions management revealed that hospitals in which two or more ward rounds of all acute medical unit patients were performed daily had a lower adjusted case fatality rate for patients with hospital stays over 7 days. Despite twice-daily consultant ward rounds of all new acute admissions and the addition of a third 4:00 pm round from Monday to Friday, only 62% of patients were seen by a consultant within 12 hours. With 67% of patients being admitted between the hours of 12:00 am and 12:00 pm, it is possible that the substitution of an evening round for one of the afternoon rounds would help increase the number of patients seen within the target time frame. This would require a change in the working pattern of the acute medicine consultants.
